# Identification and validation of stable reference genes for RT-qPCR analyses of *Kobresia littledalei* seedlings

**DOI:** 10.1186/s12870-024-04924-w

**Published:** 2024-05-11

**Authors:** Haoyang Sun, Chunping Li, Siyu Li, Jiaxin Ma, Shuo Li, Xin Li, Cai Gao, Rongchen Yang, Nan Ma, Jing Yang, Peizhi Yang, Xueqing He, Tianming Hu

**Affiliations:** 1 College of Grassland Agriculture, Northwest A&F University, Yangling, 712100 Shaanxi Province PR China; 2 College of Natural Resources and Environment, Northwest A&F University, Yangling, 712100 Shaanxi Province PR China

**Keywords:** Reference gene, RT-qPCR, *Kobresia littledalei*, Normalization, Algorithms

## Abstract

**Background:**

*Kobreisa littledalei*, belonging to the *Cyperaceae* family is the first *Kobresia* species with a reference genome and the most dominant species in Qinghai-Tibet Plateau alpine meadows. It has several resistance genes which could be used to breed improved crop varieties. Reverse Transcription Quantitative Real-Time Polymerase Chain Reaction (RT-qPCR) is a popular and accurate gene expression analysis method. Its reliability depends on the expression levels of reference genes, which vary by species, tissues and environments. However, *K.littledalei* lacks a stable and normalized reference gene for RT-qPCR analysis.

**Results:**

The stability of 13 potential reference genes was tested and the stable reference genes were selected for RT-qPCR normalization for the expression analysis in the different tissues of *K. littledalei* under two abiotic stresses (salt and drought) and two hormonal treatments (abscisic acid (ABA) and gibberellin (GA)). Five algorithms were used to assess the stability of putative reference genes. The results showed a variation amongst the methods, and the same reference genes showed tissue expression differences under the same conditions. The stability of combining two reference genes was better than a single one. The expression levels of *ACTIN* were stable in leaves and stems under normal conditions, in leaves under drought stress and in roots under ABA treatment. The expression of glyceraldehyde-3-phosphate dehydrogenase (*GAPDH*) expression was stable in the roots under the control conditions and salt stress and in stems exposed to drought stress. Expression levels of superoxide dismutase (*SOD*) were stable in stems of ABA-treated plants and in the roots under drought stress. Moreover, *RPL6* expression was stable in the leaves and stems under salt stress and in the stems of the GA-treated plants. *EF1-alpha* expression was stable in leaves under ABA and GA treatments. The expression levels of *28 S* were stable in the roots under GA treatment. In general, *ACTIN* and *GAPDH* could be employed as housekeeping genes for *K. littledalei* under different treatments.

**Conclusion:**

This study identified the best RT-qPCR reference genes for different *K. littledalei* tissues under five experimental conditions. *ACTIN* and *GAPDH* genes can be employed as the ideal housekeeping genes for expression analysis under different conditions. This is the first study to investigate the stable reference genes for normalized gene expression analysis of *K. littledalei* under different conditions. The results could aid molecular biology and gene function research on *Kobresia* and other related species.

**Supplementary Information:**

The online version contains supplementary material available at 10.1186/s12870-024-04924-w.

## Introduction

More than 70 species of *Kobresia*, a perennial herb belonging to the *Cyperaceae* family, are found mostly in the alpine ranges of the northern hemisphere and are mainly distributed in the Qinghai-Tibet Plateau in China [1]. They represent the primary year-round food supply for local grazing animals, particularly yaks, due to their nutritional qualities and large biomass [2, 3]. The *Kobresia* plants are also vital for preserving the ecological equilibrium of grasslands. *Kobresia littledalei* is the dominant species in low-lying locations around lakes, river borders and saline marsh in alpine meadows of the Qinghai-Tibet Plateau [1]. It has an underground short rhizome and the mature height is 20 to 30 cm. *K. littledalei* has evolved several ideal resistance genes after a long period of natural selection in the harsh environmental conditions of the Qinghai-Tibet Plateau, especially the genes conferring resistance to cold, radiation, drought, and strong wind stress. These genes could be mined and utilized for breeding improved crop varieties [4]. Changes in the gene expression levels are the direct biomarkers that may be used to evaluate an organism’s reaction to an altered environment [5]. Few studies have explored the molecular resistance mechanisms of *K. littledalei*. The first reference genome of the genus *Kobresia* was reported in 2020 [1], and it showed that *K. littledalei* is a diploid (2n = 2x = 58) with a 373.85-Mb assembly size. Qu et al. [6] explored the transcriptome of *K. littledalei* in response to cold stress. These studies were the beginning of understanding the resistance mechanisms of *K. littledalei* response to such harsh environments.

The introduction of quantitative reverse transcription-polymerase chain reaction (RT-qPCR) has drastically revolutionized gene expression analysis [7] due to its several benefits, including a broad dynamic range, high sensitivity, specificity, throughput, and precision [8]. However, RNA quality, integrity, reverse transcription efficiency, and amplification efficiency can influence the precision of RT-qPCR findings [9]. Hence, reference genes are frequently employed to decrease or rectify faults during target gene quantification to ensure accurate results without analytical errors [10].

Genes involved in the maintenance of basic cellular activities and those encoding proteins have frequently been used as reference genes because their products are required for cellular biological activity and may potentially be produced under any conditions [11]. These genes include the 18 S ribosomal RNA (*18 S*) gene, one of the most conserved genes in all cells [12] and the glycero-aldehyde-3-phosphate dehydrogenase (*GAPDH*) gene, a key enzyme in the carbon fixation pathways of glycolysis, gluconeogenesis, and photosynthesis [13, 14]. An ideal reference gene should have a reasonably stable and consistent expression level across cultivars, tissues, and environmental circumstances [15]. Yet, the expression of these housekeeping genes varies significantly in different experimental conditions and plant tissues [16, 17]. For instance, the conventional reference gene *ACTIN* showed the most stable expression under drought stress in garlic (*Allium sativum*), but it was not reliable under cold stress [18]. Additionally, *ACTIN* is not recommended for RT-qPCR analysis of *Miscanthus sacchariflorus* under drought, salt, and cadmium stress conditions [11]. Copper/zinc superoxide dismutase (*Cu/Zn-SOD*) was reported to be the best reference gene during seed soaking and stratification treatment of *Magnolia sieboldii*; however, it is not suitable for various organs and seeds at different developmental stages [19]. Similarly, the elongation factor 1-alpha (*EF1-alpha*) was the most stably expressed reference gene in oat (*Avena sativa*) roots under UV-B exposure, whereas PSK SIMULATOR 1-like (*PSKS1*) was the most stable expressed reference gene under high light stress [13]. Hence, it is vital to choose the most suitable reference genes for the various tissues or conditions to eliminate errors and ensure the accuracy and reliability of the data.

Therefore, screening for stable reference genes of *K. littledalei* important for revealing its molecular mechanisms of stress tolerance and gene expression processes via qRT-PCR [20]. This study investigated the expression stability of 13 potential reference genes in distinct physiological tissues (leaf, stem, and root) of *K. littledalei* plants subjected to two abiotic stimuli (salt and drought) and two exogenous hormonal treatments (abscisic acid and gibberellin). Subsequently, five normalizing algorithms (Delta-Ct, geNorm, NormFinder, BestKeeper, and RefFinder) were utilized to evaluate the expression stability of the genes. Selecting reliable reference genes is recommended for standardizing RT-qPCR data in various contexts. To verify the applicability of the selected reference genes, we selected *BSK5* (Brassinosteroid-Signaling Kinase 5) and *AP2/ERF* (APETALA2/Ethylene-Responsive Factor), involved in various plant responses under biotic or abiotic stresses [6, 21, 22], for validation. This is the first study to conduct a systematic analysis for the selection of reference genes in *K. littledalei* tissues subjected to various treatments. The results could facilitate future studies on the gene expression and molecular mechanisms of *K. littledalei*.

## Materials and methods

### Plant material, growth conditions and treatments

*Kobresia littledalei*, with a known genome, was used as the experimental plant. The Tibet Academy of Agricultural and Animal Husbandry Sciences provided mature seeds collected in 2014 from Naqu, Tibet, China, and stored at 4 °C. The seeds were surface sterilized using 75% ethanol (v/v) for 30 s and 1% sodium hypochlorite solution for 15 min. The sterilized seeds were germinated in a Petri dish (90 mm) for 30 days, sown in a pot filled with vermiculite, and moistened daily with Hoagland’s nutrient solution in an artificial climate incubator with a 25 °C / 18 °C average temperature, 70% relative humidity, and a 16 h / 8 h (light/dark) photoperiod of 1125 µM photons m^−2^ s^−1^ [23]. *Kobresia* represent typical drought-tolerant, cold-tolerant, and barren plants of the alpine meadows [24]. Thus, this study subjected one-year-old plants to different abiotic and exogenous hormone sprays as follows: (1) drought treatment; 400 mM mannitol; (2) salt treatment; 200 mM NaCl; (3) exogenous hormone treatment; 100 µM ABA and (4) 100 µM GA, respectively; and (5) control; normal conditions. Abiotic stress was induced by adding drugs to the hydroponic treatment; exogenous hormonal treatment involved spraying plants once. The hormones were dissolved in distilled water and sprayed on the plants until the droplets did not drip off. Finally, samples from different organs (leaves, stems, and roots) were collected from plants at 0, 2, 6, 9, 12, and 24 h after treatment, frozen in liquid nitrogen, and stored at -80 °C. Each experiment had three biological replicates, and each replicate contained at least two seedlings.

### RNA extraction and cDNA synthesis

Total RNA from *Kobresia* was extracted using the Eastep Super Total RNA Extraction Kit (Promega Corporation, Wisconsin, USA) following the manufacturer’s instructions. The purity and concentration of RNA were evaluated using a nanodrop2000 spectrophotometer (Thermo Fisher Scientific, MA, USA). The RNA samples had optical density (OD) ratios of 1.8-2 and > 2 for OD260 / OD280 and OD260 / OD230, respectively. Then, single-stranded complementary DNA (cDNA) was synthesized from 0.5 µg RNA of each sample using the HiScript III RT SuperMix qPCR cDNA synthesis kit (Vazyme Bio, Shanghai, China) following the manufacturer’s instructions. All the cDNA samples were diluted to 1× and stored at -20 °C for later use.

### Selection of candidate reference genes

The top ten primers from ICG (http://icg.big.ac.cn/index.php/Main_Page) [25] and other common housekeeping genes were used to select 13 candidate reference genes: Actin (*ACTIN*), glyceraldehyde-3-phosphate dehydrogenase (*GAPDH*), 18 S ribosomal RNA (*18 S*), 28 S ribosomal RNA (*28 S*), TATA box binding protein (*TBP*), eukaryotic translational elongation factor 1 alpha (*EF1-alpha*), ubiquitin (*UBQ*), alpha-tubulin (*TUA*), copper/zinc superoxide dismutase (*SOD*), ribosomal protein, large, 6 (*RPL6*), cyclophilin (*CYP*), HIS triad family protein 3 (*HIS*), and Leucyl-tRNA (*LEU*). All the 13 candidate reference genes are commonly used as housekeeping genes in model plant species. These genes were cloned according to their coding sequences (CDS) from a recently published representative first draft whole genome of *K. littledalei* (NCBI accession number: ASM1111435v1) [1]. The primers were designed on the web using the Primer 3.0 plus (http://www.primer3plus.com, accessed on August 2021) software, and then the theoretical annealing temperature of each primer was predicted accordingly. The primers were synthesized by Sangon Biotech Company (Shanghai, China), and the PCR products were resolved on 1% agarose gel. The primer data of the candidate reference genes are listed in Table 1.

**Table 1:** Candidate reference genes, amplicon characteristics, and primer sequences

Gene Symbol	Description	Genebank ID	Primer sequences forward/ Reverse (5′–3′)	Tm (℃)	Amplicon size (bp)	PCR Efficiency (%)	*R*^*2*^
***ACTIN***	ACTIN	KAF3327872.1	TGCTAGACTCGGGAGATGGTGTTAG	66	85	116.454	0.9924
			AAGTCAAGACGTAGGATTGCATGGG				
***TBP***	TATA box binding protein	KAF3321489.1	TACTCGGGTCCTGCCAACTA	64	234	130.970	0.9904
			CCGACATCACGACAACTCGA				
***28 S***	28 S ribosomal RNA	EU854168.1	GAACCATCGAGTCTTTGAAACGC	64	262	107.212	0.9800
			TCCTCGTTAGGGGATCAAACAAG				
***UBQ***	Polyubiquitin	KAF3337050.1	CGCCTGATTTATGCCGGGAAGC	67	93	113.438	0.9935
			CCTCATCAACAGGTGCAGTGTCG				
***GAPDH***	Glyceraldehyde-3-phosphate dehydrogenase	KAF3337947.1	GGAGGAGTCTGAGGGCAAAC	64	201	106.397	0.9982
			TGGCGGACTAGGTCAACAAC				
***SOD***	Copper/zinc superoxide dismutase	KAF3326232.1	GGGTGTCAAGGGCACTATTT	62	236	110.665	0.9975
			CCTCTCCAGCAGTCACATTTC				
***RPL6***	Ribosomal protein L6	KAF3341553.1	CCCTTGTAAACTTCAGGTGGTTTG	63	201	117.684	0.9960
			CAAGGCTAGAACTGAATCAGCAG				
***HIS***	HIS triad family protein 3	KAF3330657	GAGTAGACTGTCGGTTTTGAGCT	63	241	117.200	0.9973
			CGGGATGATAATGATGTGGGTTG				
***CYP***	Cyclophilin	KAF3336147.1	GTGATGGAGTTGTACGCCGA	64	201	114.856	0.9971
			GCCGTAAATGGATTCACCGC				
***TUA***	Tubulin-Alpha	KAF3323052.1	CTCTTCCATCCTGAGCAACTCAT	64	210	115.927	0.9984
			CTCAAGGAGGAGAGAACCAAGAC				
***18 S***	18 S ribosomal RNA	JF715288.1	CCGTGAACCATCGAGTCTTT	62	272	104.919	0.9983
			CGGCATGCTCCTCGTTAG				
***EF1-alpha***	Elongation factor-1alpha	KAF3331716.1	TTGAGACCACCAAGTACTACTGC	64	223	99.718	0.9988
			TTGTTGCAACAGCAGATCATCTG				
***LEU***	Leucyl-tRNA	EU854199.1	GGTTCAAGTCCCTCTATCCCC	62	383	95.106	0.9935
			TCTTGTGGATCACTCGAGTAGA				

The amplification efficiency (E) and correlation coefficients (R^2^) were calculated using a standard curve based on tenfold serial dilutions of a mixture of the synthesized cDNA over six dilution points, starting from 1000 ng µL^−1^. The threshold cycle (Ct) was measured automatically, and the corresponding RT-qPCR efficiency (E) for each gene was determined from the given slope (Table 1).

### RT-qPCR conditions

After the quality tests of the primers, RT-qPCR was performed in 96-well plates in the Light Cycler480 real-time PCR System (Roche Molecular Systems, Mannheim, Germany). The reaction mixture contained 2.6 µL ultrapure water, 5 µL ChamQ SYBR qPCR Master Mix (Vayzme Bio, Shanghai, China), 2 µL cDNA, 0.2 µL forward primer (10µM), and 0.2 µL reverse primer (10 µM). The program involved denaturation for 1 min at 94 °C, 40 cycles of 10 s at 94 °C, and 30 s at 62 °C. Melt curves were obtained by heating the sample from 60 to 95 °C at a rate of 1.0 °C·s^−1^. Each treatment had three biological and two technical replicates.

### Analysis of gene expression Stability

Raw qPCR data were collected using LightCycler® 96 software v. 1.1 (Roche Molecular Systems, Mannheim, Germany). The reference genes were ranked across all tissues and tissue combinations using the delta-Ct method [26], geNorm [7], NormFinder [27], BestKeeper [28], and RefFinder [29]. The geNorm program calculates the average expression stability measurement (M) value according to the pairwise variation between two sequences, eliminating the genes that show the worst expression stability in a stepwise manner. NormFinder calculates the stability value based on variance analysis, overcoming the limitations of geNorm, which cannot discriminate between coregulated genes. BestKeeper evaluates expression stability by calculating the standard deviation (SD) and the percentage covariance (CV). BestKeeper and geNorm are based on pairwise comparison; hence, they have the same limitations regarding coregulated genes [30]. However, the Delta-Ct method determined the ranks after pairwise comparisons of gene sets. The reference gene with the lowest SD had the most stable expression. The geNorm, BestKeeper, Delta-Ct, and NormFinder analyses were performed using the ctrlGene [31] and NormqPCR [32] packages in R 4.2.1. Finally, RefFinder (http://www.leonxie.com/refer encegene.php) was used to calculate the comprehensive ranks based on the geometric mean values from the results of the other four methods.

### Validation of reference genes

Brassinosteroid-signaling kinase 5 (*BSK5*) and APETALA2/Ethylene-Responsive Factor (*AP2/ERF*) were selected as target genes to confirm the reliability of the candidate reference genes. The top two most stable genes normalized *BSK5* and *AP2/ERF* expression stability, and RefFinder identified the most unstable gene across each treatment, tissue, and sample. The best primer pairing, ranked by geNorm, was also used to normalize the target genes. The samples were collected at the same time as described above under drought and salt stress, and the RT-qPCR amplification conditions were the same as described above. The 2^−ΔΔCt^ method [33] was used to calculate the expression of target genes in each condition. Table S1 showed the primer sequences for *BSK5* and *AP2/ERF*.

## Results

### Primer specificity and amplification efficiency of candidate reference genes

**Fig. 1:**
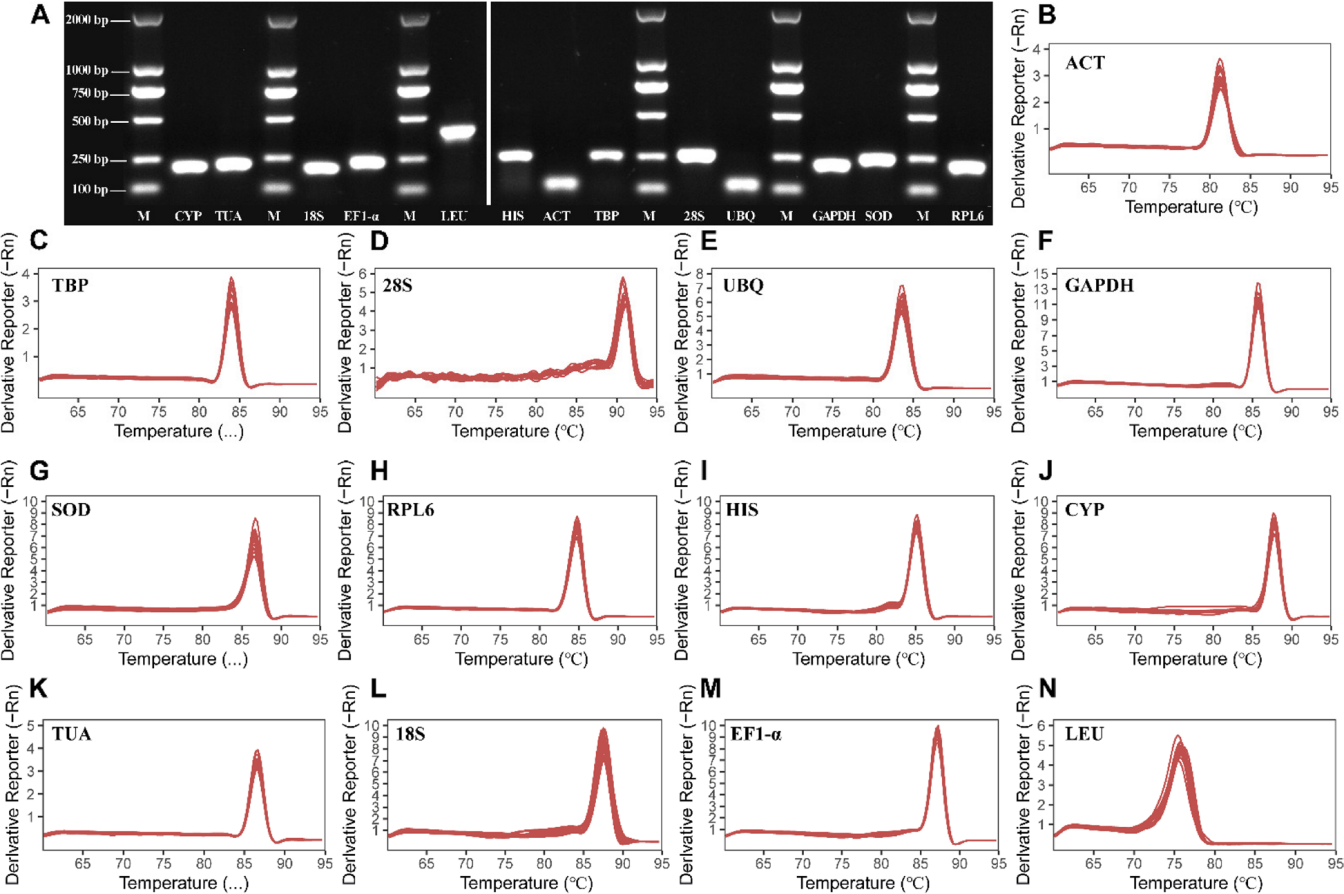
Specificity of primers and amplificon lengths, and melt curves of qPCR amplification of 13 candidate reference genes. **A** Specific product length of each reference gene was indicated after 1% agarose gel electrophoresis. Marker represents Marker DL2000. The image is an adjacent lane of the same gel (1%), and the image size is cropped; **B**-**N** were the melt curves of reference genes

Table 1 summarizes the thirteen putative housekeeping genes, including their complete names and GeneBank accession numbers. The 1% agarose gel electrophoresis findings demonstrated that each primer had a single, bright band (Fig. 1A). Furthermore, the melting curves for all candidate genes exhibited single peaks (Fig. 1B-N) and dissolving curves demonstrated amplification efficiencies ranging from 95.106% to 130.970%, with 0.9800 to 0.9988 correlation coefficient (R^2^) (Table 1). The preceding results suggested that the primers had reasonable specificity. Notably, the melting temperature of *LEU* primers was < 80℃; hence, the *LEU* gene was rejected from further investigation.

### Relative expression of candidate genes in all samples

**Fig. 2:**
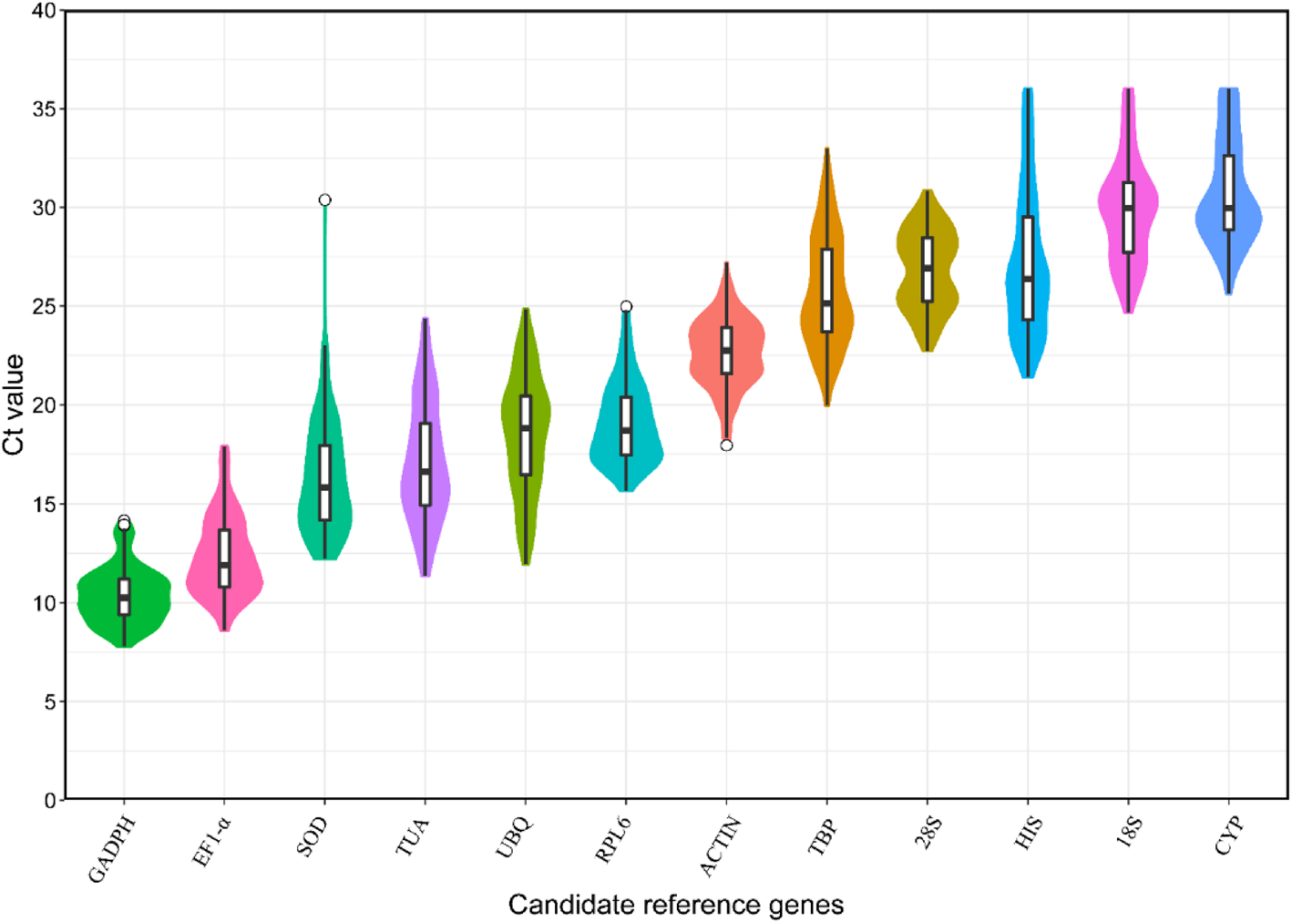
Violin plot analysis of Ct value of 12 candidate reference genes in all samples. The boxes indicate the 25th and 75th percentiles. The line across the box represents the median. The whisker shows the maximum and minimum values, respectively. The circle out of the violin represents the outside values

Figure 2 shows the transcriptional abundance of 12 housekeeping genes in 30 samples under five distinct environmental circumstances (mannitol, NaCl, ABA, GA, and normal conditions) and six different time treatments (0, 2, 6, 9, 12, 24 h). The results revealed that the mean Ct values for all reference genes ranged from 10.38 to 30.72, indicating a disparity in their expression levels. *GAPDH* had the lowest mean Ct value (10.38), correlating to the greatest expression level. However, *CYP* had the highest mean Ct value and the lowest expression level. The standard deviation (SD) of the Ct indicated the variation in gene expression levels across the samples (Fig. 2). *GAPDH* had the lowest SD (1.36), indicating that it is more stable under diverse settings. Nonetheless, the expression of *HIS* varied greatly among the samples, indicated by its SD value of 3.49.

### Estimation of stability by Delta-Ct

**Fig. 3:**
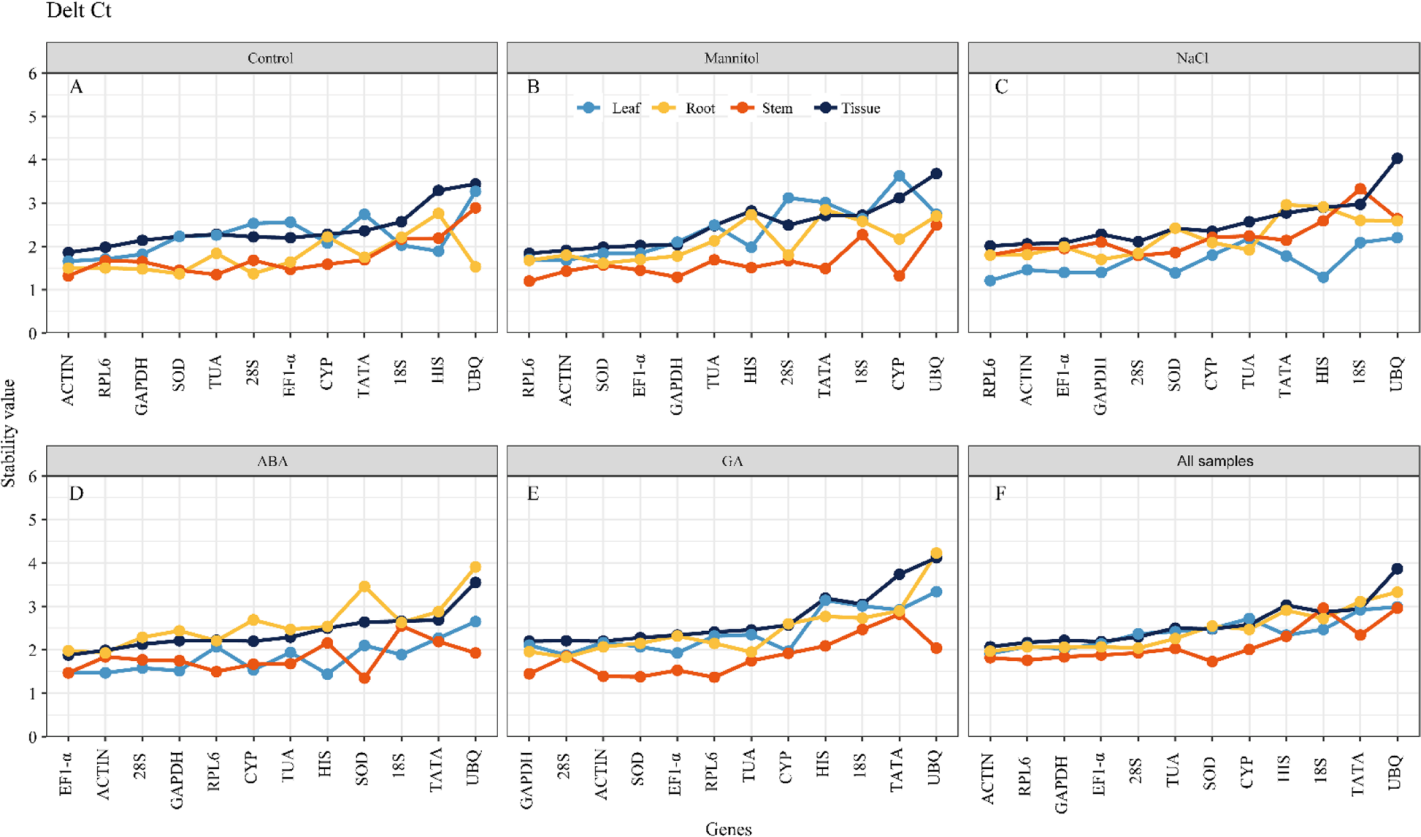
Expression stability rankings of 12 candidate reference genes using the Delta-Ct

The relatively low Delta-Ct values among the putative housekeeping genes suggested a relatively strong gene expression. The Delta-Ct technique results showed that *ACTIN* is the most stable reference gene across the treatments and tissues, including leaf tissue of the control (1.66) and drought conditions (1.68), stem tissue of the control (1.32), and root tissue under ABA treatment (1.93) (Fig. 3). *RPL6* was the most stable reference gene in stem tissue under 400 mM mannitol stress (1.20) and exhibited excellent stability in leaf tissue under NaCl conditions, and stem tissue under GA treatment (1.21 and 1.37). Nevertheless, *28 S* was the most stable reference gene in the GA-treated leaf and root tissues and NaCl-treated stem tissue. *SOD* was a good reference gene under control and mannitol conditions in root tissues and was stable in ABA-treated stem tissues. *HIS* was the most stable reference gene in ABA-treated leaves (1.44), while *GAPDH* was the most stable reference gene in salt-stressed roots (1.70). *ACTIN* was the most stable reference gene in each tissue under control conditions. Under abiotic stress (mannitol and NaCl), *RPL6* was the most stable reference gene.

Further, *EF1-alpha* (ABA treatment) and *GAPDH* (GA treatment) were the most stable genes under hormone treatment. *ACTIN* was the most stable reference gene for all leaf and root tissues (1.91 and 1.97, respectively) and all samples (2.07), whereas *SOD* was the most stable reference gene for all stem tissues (1.73). (Fig. 3F). The greatest delta-Ct value was observed for *UBQ* (the value of all samples was 3.87), suggesting that it was the most unstable reference gene under all circumstances.

### Estimation of stability by geNorm analysis

**Fig. 4:**
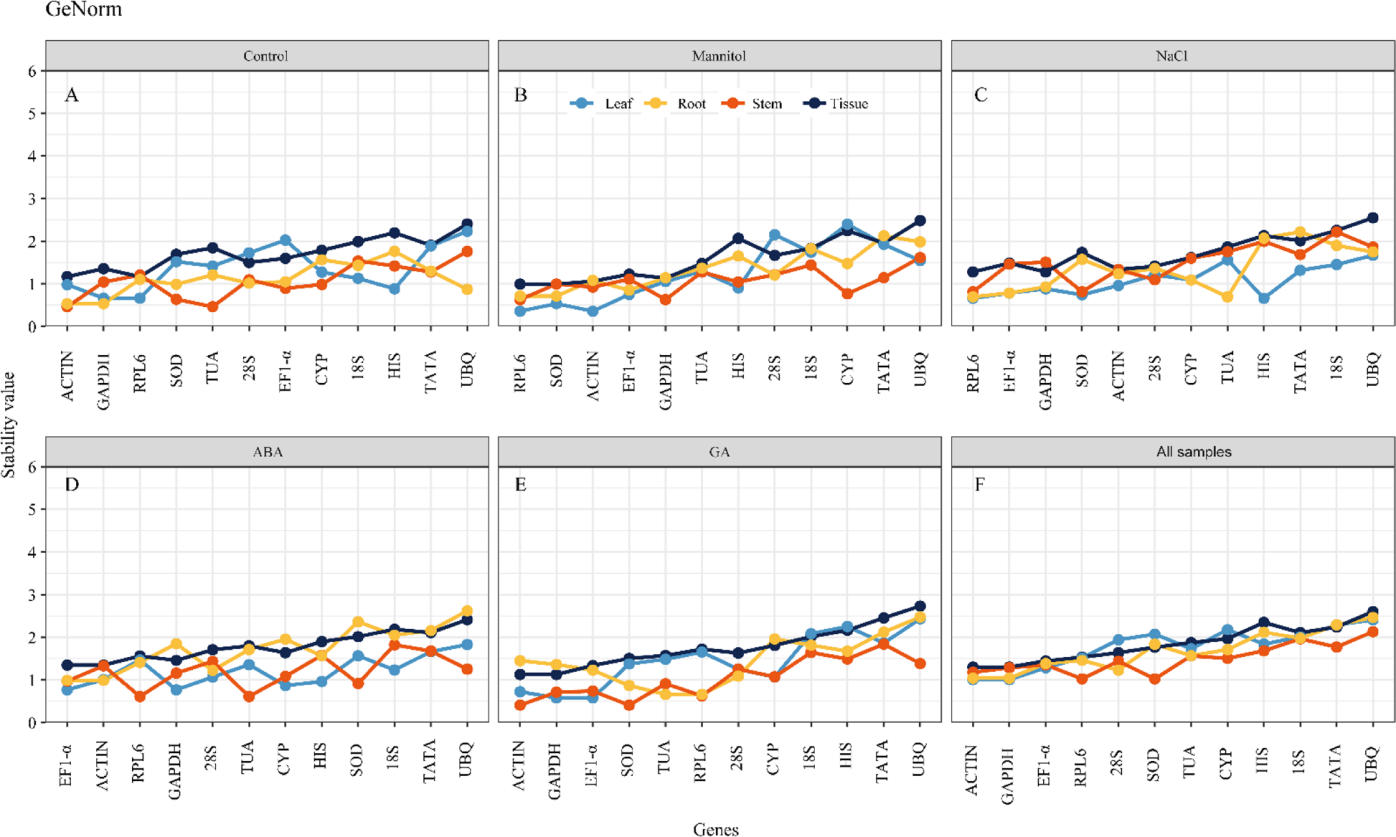
Expression stability rankings of 12 candidate reference genes using the geNorm

The geNorm algorithm was applied to all three tissues and their combinations to determine the stability of the 12 housekeeping genes under various conditions (mannitol, NaCl, ABA, GA, and normal conditions). In this technique, M represents the gene expression stability ranking; an M number < 1.5 is considered within the gene stability range. The lower the M value, the greater the gene stability [7]. Candidate reference genes with the most stable expression differed between tissues under stress or hormone treatments (Fig. 4). *ACTIN* and *RPL6* were the top two stable reference genes of *K. littledalei* in all tissues under normal conditions (M number = 1.168). In leaf tissue, *GAPDH* and *RPL6* were stable (0.664); *ACTIN* and *TUA* in stem tissue (0.463); and *ACTIN* and *GAPDH* in root tissue (0.533) (Fig. 4A). *SOD* and *RPL6* were stable under drought stress (0.994), *ACTIN* and *RPL6* in leaf (0.358), *GAPDH* and *RPL6* in stem (0.630), and *SOD* and *RPL6* in root (0.713) (Fig. 4B). In the salt stressed group, *GAPDH* and *RPL6* were the most stable housekeeping genes (1.278) in the leaf, *RPL6* and *HIS* (0.661) in the stem, *SOD* and *RPL6* (0.813) in the root tissue, and *RPL6* and *TUA* (0.694) (Fig. 4C). The top two genes under ABA and GA treatment were *ACTIN* and *EF1-alpha* (1.346), and *ACTIN* and *GAPDH* (1.127), respectively. *GAPDH* and *EF1*-*alpha* were the most stable genes in the leaf tissue of the ABA (0.767) and GA (0.578) treatment groups. *RPL6* and *TUA* were the most stable reference genes in the tissues of the ABA-treated stem (0.610) and GA-treated root (0.659). The best reference genes for the ABA-treated root and GA-treated stem tissues were *ACTIN* and *EF1-alpha* (0.988), and *ACTIN* and *SOD* (0.409), respectively (Fig. 4D, E). *ACTIN* and *GAPDH* had the most consistent expressions across multiple tissues and treatments (1.298) (Fig. 4F). Similarly, these two genes showed the lowest M value in the leaf (1.007) and root (1.040) under all tissue circumstances. Nevertheless, *SOD* and *RPL6* exhibited steady expression in all stem samples (1.024). *UBQ*, which was consistently in the final place neither in single tissues nor tissue combinations, was unsatisfactory for normalizing RT-qPCR results for *K. littledalei*.

**Fig. 5:**
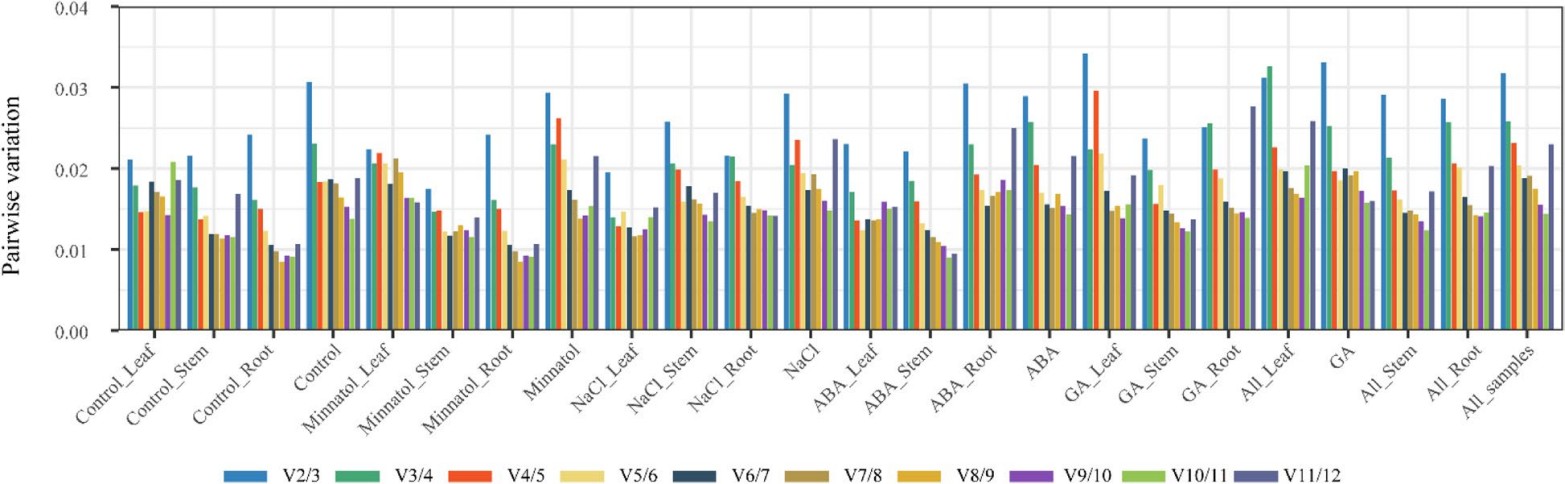
Pairwise variation (Vn/Vn + 1) analysis of the optimal number among ten candidate reference genes in different experimental sets

As required by the Minimum Information for Publication of Quantitative Real-time PCR Studies (MIQE) requirements [34], the pairwise variation V (Vn/Vn + 1) of the normalization factor was also determined (Fig. 5). All group pairwise variations were below the general assumption cutoff of 0.15, indicating that the two reference genes were adequate for normalizing RT-qPCR data. Hence, the M and Vn + 1 values provided by geNorm identified *ACTIN* and *GAPDH* as the most stable genes among all samples and the leaf and root tissues of all treatments. In contrast, *SOD* and *RPL6* were the most stable genes in stem tissues under all circumstances. *ACTIN* and *RPL6*, *SOD* and *RPL6*, *GAPDH* and *RPL6*, *ACTIN* and *EF1-alpha*, and *ACTIN* and *GAPDH* were the most stable combinations during normal conditions, mannitol stress, NaCl stress, and ABA and GA treatment, respectively.

### Estimation of stability by NormFinder

**Fig. 6:**
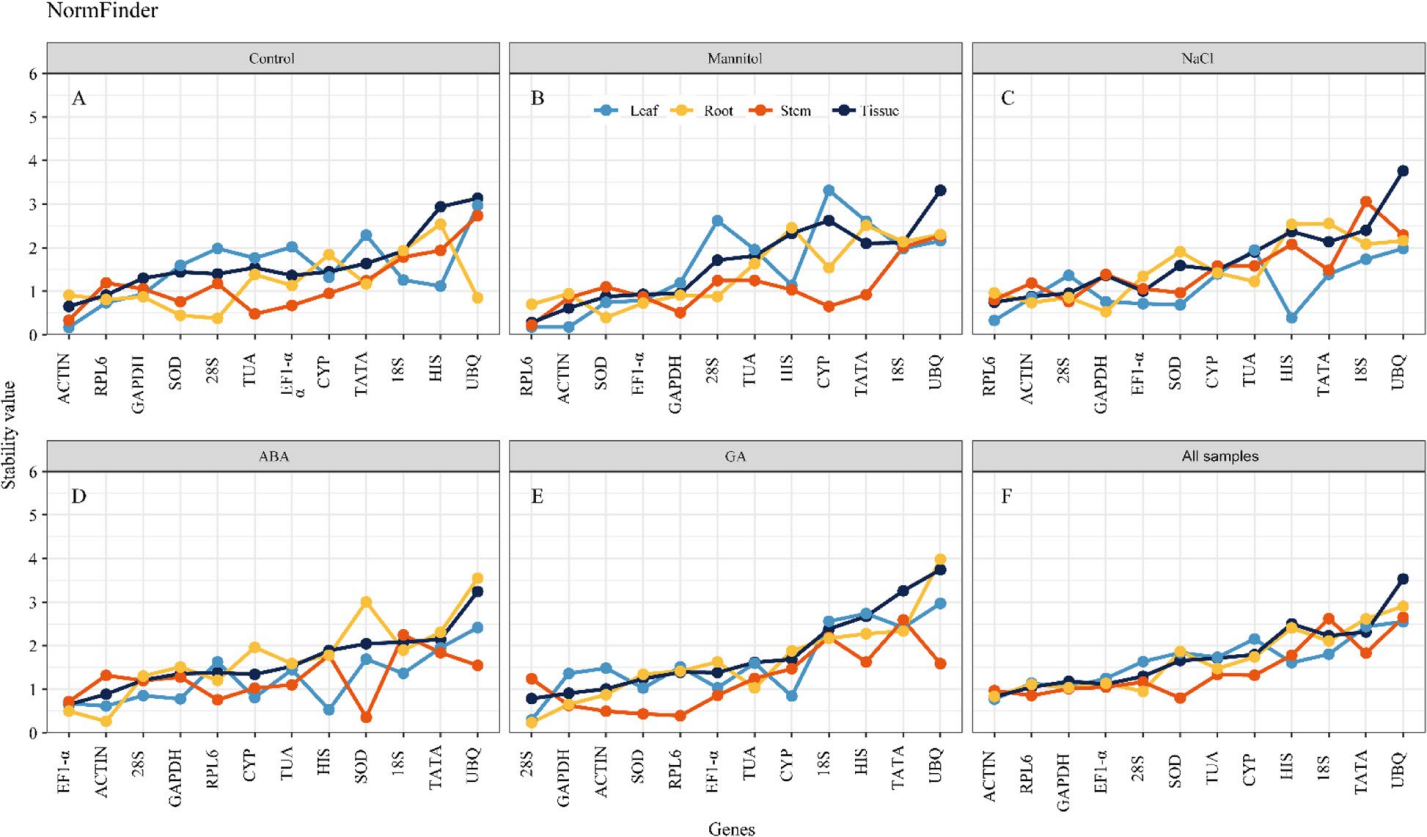
Expression stability rankings of 12 candidate reference genes using the NormFinder.

The lower the NormFinder stability values, the more stable the reference genes. Thus, *ACTIN* was the most stable reference gene in leaf and stem tissues under normal conditions, whereas *28 S* was the most stable in root tissue (Fig. 6A). Under mannitol treatment, *RPL6* was the ideal reference gene in both leaf and stem tissues, and *SOD* was ideal in root tissue (Fig. 6B). Under salt stress, *RPL6* was the most stable gene in the leaf, *28 S* in the stem, and *GAPDH* in the root (Fig. 6C). *HIS* was the most stable gene in leaf tissue, whereas *SOD* and *ACTIN* were the most stable genes in stem and root tissue, respectively (Fig. 6D). *28 S* was the most stable housekeeping gene in *K. littledalei* leaf and root tissues under GA conditions, while *RPL6* was most stable in the stem (Fig. 6E). Moreover, the tissue stability value of each treatment revealed *ACTIN* and *RPL6* as the best reference genes under control conditions, and that they perform well during drought and salt stress. *EF1-alpha* and *ACTIN* were the best candidate reference genes under the ABA condition, whereas *28 S* and *GAPDH* were most stable under GA treatment. *ACTIN* exhibited the lowest stability value (0.827) across all samples, stem, and root tissue groups (0.772 and 0.837, respectively). *SOD*, *RPL6*, and *ACTIN* were the top three stable reference genes in all stem tissue (Fig. 6F). Meanwhile, *UBQ* had the lowest stability in over half of the examined population (Fig. 6).

### Estimation of stability by BestKeeper

**Fig. 7:**
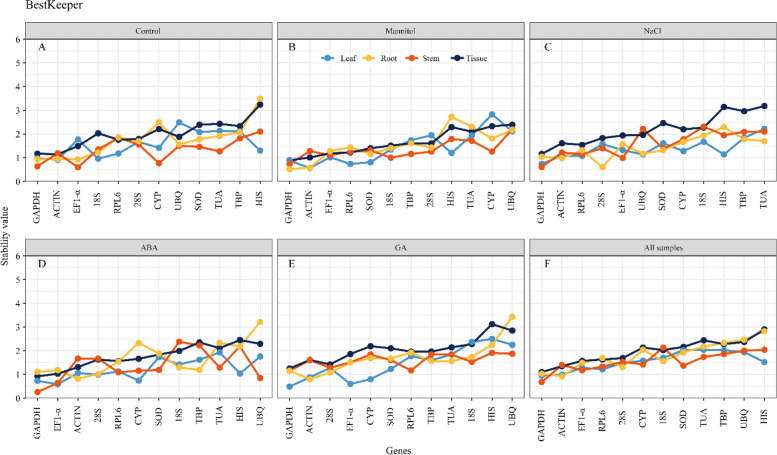
Expression stability rankings of 12 candidate reference genes using the BestKeeper.

In the BestKeeper analysis, the candidate reference genes were ordered based on the standard deviations of the original Ct values of each candidate gene under various settings. Genes with < 1.5 standard deviation values were considered the most stable—furthermore, the smaller the SD value, the greater the gene stability. The results of the stability study of samples from various treatments indicated *GAPDH* (1.10) as the best suitable reference gene across nearly all tissues and treatments (Fig. 7). *ACTIN* exhibited excellent stability in the control (1.15) and mannitol-treated (0.57) leaf tissues, and ABA- (0.82) and GA-treated (0.80) root samples. Under normal conditions, *EF1-alpha* was very stable in the stem (0.60) but exhibited poor stability in the stem (1.51) and root (1.50) under GA treatment. *28 S* was the most stable reference gene in NaCl-treated root samples (0.60) and the most variable. *GAPDH* was the most stable reference gene across all samples (1.10), all leaf (0.95), and stem (0.68) tissues, and *ACTIN* in the root (0.92). The SD values of the *HIS*, *UBQ*, and *TUA* were the highest of all the reference genes, demonstrating the instability of these three genes (the average values were 2.27, 2.09, and 2.02, respectively).

### Comprehensive ranking analysis

RefFinder is an exhaustive algorithm incorporating Delta-Ct, geNorm, NormFinder, and BestKeeper analysis tools. Table 2 displays the RefFinder results, which indicated that *ACTIN* was the optimal reference gene in the leaf (1.41) and stem (1.41) tissues under normal circumstances, leaf tissues (1.19) under mannitol treatment, and root tissues (1.00) responding to ABA treatment. In mannitol-treated stem and root tissues, *GAPDH* (1.41) and *SOD* (1.32) were the optimum reference genes. *RPL6* was the suitable reference gene in leaf and stem samples treated with NaCl (1.32 and 1.86, respectively). *EF1-alpha* was the most stable reference gene in leaf tissues treated with spraying hormone (ABA was 1.73 and GA was 2.00, respectively). Further, *SOD* (2.06) was the optimal reference gene in the ABA-treated stem, followed by *EF1-alpha* (2.38). In GA-treated stem tissue, *RPL6* was the most stable gene (1.57), and *28 S* was the most stable gene in root tissue (1.68). *RPL6* was the optimal reference gene for drought- (1.41) and salt- (1.19) stressed samples, whereas *ACTIN* was appropriate for normal circumstances (1.00). Besides, the best reference genes under ABA and GA treatments were *EF1-alpha* (1.19) and *GAPDH* (1.19), respectively. *ACTIN* and *GAPDH* were the two most stable reference genes in all leaf (1.19 and 1.41, respectively) and root samples (1.00 and 2.06, respectively). In contrast, *SOD* (1.41) and *RPL6* (1.86) were the most stable reference genes in all stem samples. *UBQ* was determined to be the least stable reference gene in most samples. Based on the total number of samples across all circumstances, RefFinder rated the stability of the 12 candidate reference genes as follows: *ACTIN* > *GAPDH* > *RPL6* > *EF1-alpha* > *28 S* > *SOD* > *CYP* > *TUA* > *18 S* > *TBP* > *HIS* > *UBQ*.

**Table 2:** Comprehensive stability rankings of 12 candidate reference genes

Treatment	Tissue	Rank											
		1	2	3	4	5	6	7	8	9	10	11	12
Control	Leaf	***ACTIN*** **(1.41)**	*RPL6* (2.00)	*GAPDH* (2.28)	*HIS* (3.94)	*18 S* (3.98)	*CYP* (6.00)	*SOD* (7.71)	*TUA* (8.38)	*28 S* (8.45)	*EF1-α* (9.69)	*TBP* (10.49)	*UBQ* (12.00)
	Stem	***ACTIN*** **(1.41)**	*TUA* (2.11)	*EF1-α* (2.63)	*SOD* (3.98)	*CYP* (4.40)	*GAPDH* (4.56)	*28 S* (7.71)	*RPL6* (8.38)	*18 S* (9.01)	*TBP* (9.24)	*UBQ* (10.84)	*HIS* (10.98)
	Root	***GAPDH*** **(2.34)**	*SOD* (2.74)	*28 S* (2.78)	*ACTIN* (2.91)	*EF1-α* (4.14)	*UBQ* (4.36)	*RPL6* (5.38)	*18 S* (8.14)	*TBP* (8.71)	*TUA* (8.74)	*CYP* (10.74)	*HIS* (12.00)
	All	***ACTIN*** **(1.00)**	*RPL6* (2.00)	*GAPDH* (2.71)	*EF1-α* (3.94)	*28 S* (4.73)	*SOD* (6.82)	*CYP* (7.24)	*TUA* (8.66)	*TBP* (9.00)	*18 S* (9.15)	*UBQ* (10.09)	*HIS* (11.24)
Mannitol	Leaf	***ACTIN*** **(1.19)**	*RPL6* (1.41)	*SOD* (3.00)	*EF1-α* (4.23)	*HIS* (5.23)	*GAPDH* (5.42)	*TUA* (7.45)	*18 S* (7.97)	*UBQ* (9.19)	*TBP* (9.46)	*28 S* (10.74)	*CYP* (12.00)
	Stem	***GAPDH*** **(1.41)**	*RPL6* (1.50)	*CYP* (3.71)	*ACTIN* (4.76)	*EF1-α* (4.79)	*TBP* (5.83)	*18 S* (7.18)	*SOD* (7.33)	*HIS* (7.54)	*28 S* (8.13)	*TUA* (10.00)	*UBQ* (12.00)
	Root	***SOD*** **(1.32)**	*RPL6* (2.30)	*GAPDH* (3.16)	*EF1-α* (3.22)	*ACTIN* (3.94)	*28 S* (5.42)	*CYP* (7.97)	*18 S* (7.98)	*TUA* (8.10)	*UBQ* (10.24)	*HIS* (10.69)	*TBP* (10.84)
	All	***RPL6*** **(1.41)**	*ACTIN* (2.21)	*SOD* (2.59)	*GAPDH* (3.16)	*EF1-α* (3.94)	*28 S* (6.74)	*TUA* (6.90)	*18 S* (7.90)	*TBP* (8.24)	*HIS* (10.00)	*CYP* (11.00)	*UBQ* (12.00)
NaCl	Leaf	***RPL6*** **(1.32)**	*HIS* (2.11)	*GAPDH* (3.34)	*SOD* (3.95)	*ACTIN* (4.56)	*EF1-α* (4.6)	*CYP* (7.64)	*28 S* (7.74)	*TBP* (8.63)	*UBQ* (9.12)	*18 S* (10.00)	*TUA* (11.24)
	Stem	***RPL6*** **(1.86)**	*28 S* (2.06)	*SOD* (2.59)	*EF1-α* (3.56)	*GAPDH* (3.83)	*ACTIN* (4.47)	*CYP* (7.48)	*TBP* (7.71)	*TUA* (9.24)	*HIS* (9.69)	*UBQ* (10.74)	*18 S* (12.00)
	Root	***GAPDH*** **(1.86)**	*RPL6* (2.51)	*ACTIN* (2.91)	*28 S* (3.03)	*TUA* (3.87)	*EF1-α* (5.24)	*CYP* (6.65)	*SOD* (7.44)	*UBQ* (7.54)	*18 S* (9.97)	*HIS* (11.24)	*TBP* (11.47)
	All	***RPL6*** **(1.19)**	*GAPDH* (2.24)	*ACTIN* (2.45)	*28 S* (3.72)	*EF1-α* (4.16)	*CYP* (6.24)	*SOD* (7.45)	*TUA* (8.85)	*TBP* (9.24)	*UBQ* (10.09)	*18 S* (10.16)	*HIS* (10.24)
ABA	Leaf	***EF1-α*** **(1.73)**	*HIS* (2.11)	*GAPDH* (2.38)	*ACTIN* (3.31)	*CYP* (3.87)	*28 S* (5.42)	*18 S* (7.24)	*RPL6* (8.45)	*TUA* (8.85)	*SOD* (10.00)	*TBP* (10.46)	*UBQ* (11.74)
	Stem	***SOD*** **(2.06)**	*EF1-α* (2.38)	*RPL6* (2.45)	*TUA* (3.64)	*GAPDH* (3.98)	*CYP* (4.47)	*UBQ* (6.42)	*28 S* (7.42)	*ACTIN* (8.24)	*HIS* (10.00)	*TBP* (11.00)	*18 S* (12.00)
	Root	***ACTIN*** **(1.00)**	*EF1-α* (2.00)	*28 S* (3.13)	*RPL6* (3.98)	*GAPDH* (4.79)	*HIS* (6.85)	*TUA* (6.98)	*18 S* (7.67)	*TBP* (8.41)	*CYP* (8.97)	*SOD* (10.16)	*UBQ* (12.00)
	All	***EF1-α*** **(1.19)**	*ACTIN* (1.86)	*GAPDH* (2.94)	*28 S* (4.05)	*CYP* (4.68)	*RPL6* (4.9)	*TUA* (7.45)	*SOD* (8.45)	*HIS* (8.85)	*18 S* (9.69)	*TBP* (10.74)	*UBQ* (11.47)
GA	Leaf	***EF1-α*** **(2.00)**	*GAPDH* (2.24)	*28 S* (2.34)	*CYP* (2.91)	*SOD* (4.36)	*ACTIN* (4.56)	*RPL6* (7.48)	*TUA* (7.97)	*TBP* (8.45)	*18 S* (10.24)	*HIS* (11.24)	*UBQ* (11.47)
	Stem	***RPL6*** **(1.57)**	*SOD* (2.21)	*ACTIN* (2.82)	*GAPDH* (2.83)	*EF1-α* (4.73)	*28 S* (5.63)	*TUA* (7.09)	*CYP* (7.74)	*18 S* (9.03)	*UBQ* (9.46)	*HIS* (10.47)	*TBP* (11.17)
	Root	***28 S*** **(1.68)**	*TUA* (2.51)	*ACTIN* (3.03)	*GAPDH* (3.22)	*RPL6* (4.36)	*SOD* (4.79)	*EF1-α* (5.6)	*CYP* (8.46)	*18 S* (9.00)	*TBP* (9.45)	*HIS* (9.69)	*UBQ* (12.00)
	All	***GAPDH*** **(1.19)**	*ACTIN* (2.06)	*28 S* (2.45)	*EF1-α* (4.16)	*SOD* (4.60)	*RPL6* (5.96)	*TUA* (6.65)	*CYP* (8.24)	*18 S* (9.24)	*TBP* (9.45)	*HIS* (10.47)	*UBQ* (11.74)
All samples	Leaf	***ACTIN*** **(1.19)**	*GAPDH* (1.41)	*RPL6* (3.22)	*EF1-α* (3.72)	*HIS* (5.48)	*28 S* (5.96)	*TUA* (7.36)	*18 S* (8.00)	*CYP* (9.15)	*SOD* (9.46)	*TBP* (10.74)	*UBQ* (11.17)
	Stem	***SOD*** **(1.41)**	*RPL6* (1.86)	*GAPDH* (2.83)	*ACTIN* (3.41)	*EF1-α* (3.98)	*28 S* (6.24)	*CYP* (6.74)	*TUA* (8.00)	*HIS* (9.46)	*TBP* (9.74)	*18 S* (11.24)	*UBQ* (11.47)
	Root	***ACTIN*** **(1.00)**	*GAPDH* (2.06)	*28 S* (2.45)	*EF1-α* (4.47)	*RPL6* (4.68)	*TUA* (6.64)	*CYP* (7.24)	*SOD* (7.74)	*18 S* (7.77)	*HIS* (10.47)	*TBP* (10.74)	*UBQ* (11.74)
	All	***ACTIN*** **(1.19)**	*GAPDH* (2.00)	*RPL6* (2.83)	*EF1-α* (3.00)	*28 S* (5.00)	*SOD* (6.45)	*CYP* (7.74)	*TUA* (7.84)	*18 S* (8.13)	*TBP* (9.74)	*HIS* (11.24)	*UBQ* (11.47)

### Validation of the candidate reference genes

**Fig. 8:**
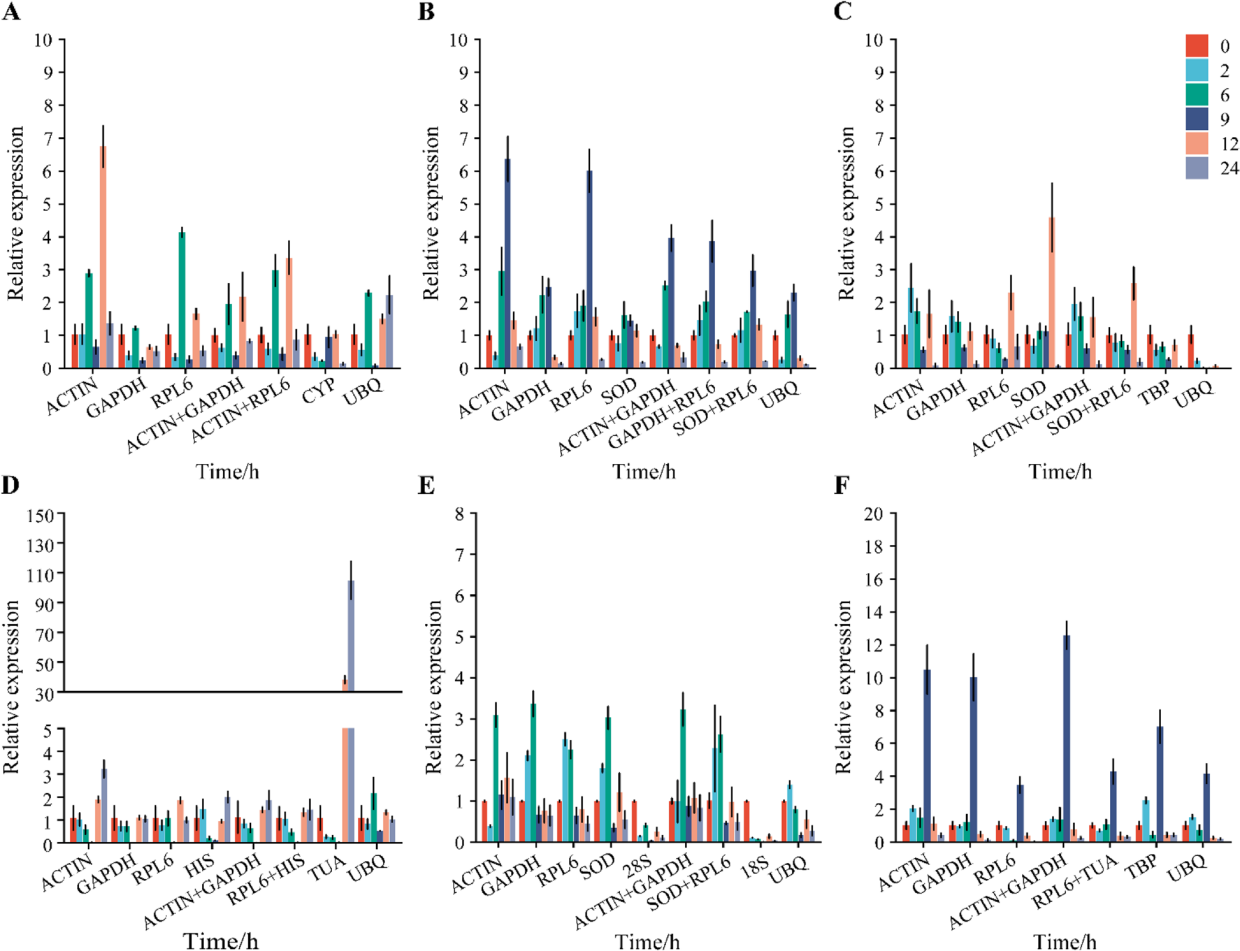
Relative expression level of *BSK* in *Kobresia littledalei* under mannitol and NaCl stress using selected reference genes. The results were normalized using the selected stable reference genes (alone or in combination) and the unstable genes in sample sets (alone or in combination) across treatment with A-C mannitol treatment in leaves, stems, and roots; D-F NaCl treatment in leaves, stems, and roots. The bars indicate the standard Deviation (± SD) evaluated from three biological replicates

**Fig. 9:**
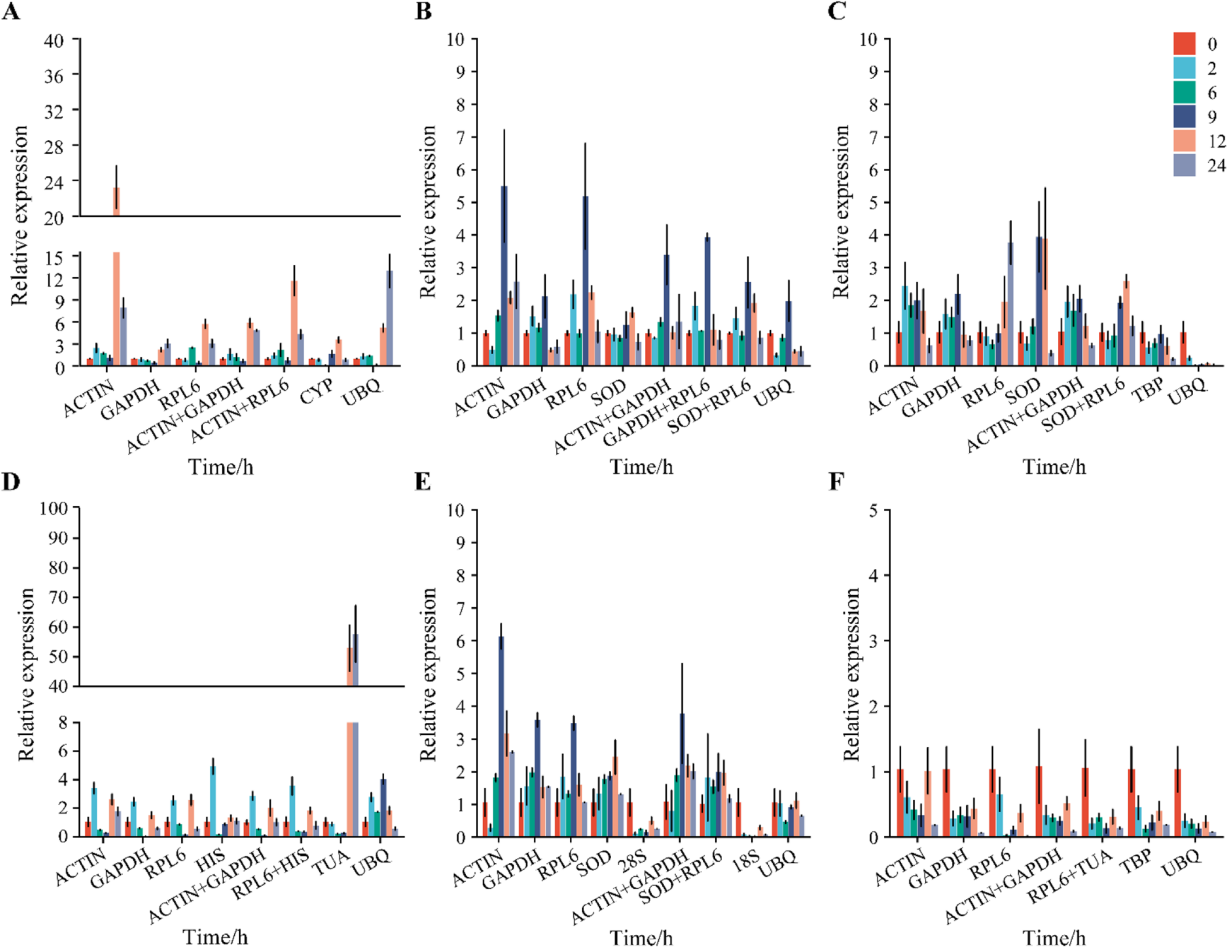
Relative expression level of *AP2/ERF* in *Kobresia littledalei* under mannitol and NaCl stress using selected reference genes. The results were normalized using the selected stable reference genes (alone or in combination) and the unstable genes in sample sets (alone or in combination) across treatment with A-C mannitol treatment in leaves, stems, and roots; D-F NaCl treatment in leaves, stems, and roots. The bars indicate the standard Deviation (± SD) evaluated from three biological replicates

The expression of *BSK* and *AP2/ERF* was normalized by single or multiple reference genes to test the reliability of selected reference genes during the response of *K. littledalei* to drought and salt stress during 0 to 24 h (Figs. 8 and 9). In this article, the top two, stable and the least stable reference genes from mannitol- and NaCl-stressed individual tissues (leaf, stem, root) and all conditions are listed in Table 2. The best combination of stable reference genes was used for normalization. The combinations included *ACTIN* with *RPL6* for mannitol-treated leaf, *GAPDH* with *RPL6* for stem, *SOD* with *RPL6* for root; *RPL6* with *HIS*, *SOD* with *RPL6*, *RPL6* with *TUA* for NaCl-treated leaf, stem and root; *ACTIN* with *GAPDH* for all leaf, root tissues and all combined samples; and *SOD* with *RPL6* for all stem samples. The expression patterns of the two target genes differed in the three plant tissues exposed to drought or salt stress. The *BSK* and *AP2/ERF* expression levels were comparable when the top two reference genes were used to standardize the data. However, the expression of the target gene that was normalized using a combination of the top-ranked stable reference genes remained lower than those of the top two stable reference genes alone. The lowest-ranked reference genes (*CYP* for mannitol-treated leaf, *UBQ* for the stem, *TBP* for root; *TUA*, *18 S*, and *TBP* for NaCl-treated leaf, stem and root, respectively; *UBQ* for all leaf, stem, root tissues and all combined samples) were not optimal for normalizing data, resulting in varied *BSK* or *AP2/ERF* expression levels compared to the top-ranked genes. The divergence was more pronounced in NaCl-treated leaves when the lowest-ranked *TUA* was applied. For instance, the relative expression of *BSK* in leaves under NaCl stress at 24 h was 0.99 and 1.99 when normalized to *RPL6* and *HIS,* and 104.97 when normalized to *TUA*, respectively. *AP2/ERF* normalization also revealed the variance of the relative expression level. Moreover, the reference genes from individual tissues of each treatment could more accurately reflect the expression pattern of the target gene than the general primers selected from all treatments. Likewise, the relative gene expression was more accurate when two top-ranked reference genes were used to normalize the relative expression of *BSK* or *AP2/ERF* than when using a single gene. This pattern held true for all examined treatments and tissues. When the lowest-ranked gene was utilized alone for normalization, the expression of the target gene seemed unnaturally raised.

## Discussion

The Qinghai-Tibet Plateau is a significant research target for studying the response of the alpine grassland ecosystems to climate change and human activities [35–37] and the degradation and restoration of alpine steppes and meadows [38–40]. Furthermore, the *Kobresia* plants on the Tibetan Plateau are a major source of stress-resistance genes [6]. Thus, the study of gene expression patterns is the basis for understanding the early responses of plants to stress [41]. Quantitative PCR is one of the most precise techniques for analyzing the expression of various genes. The technique is highly dependable, sensitive, and dependent on the selection of reference genes for normalization [34]. Therefore, the technique requires an ideal internal control that is consistently expressed under all experimental conditions, tissues, and developmental stages of the organism to reduce or prevent experimental errors and data misinterpretation. However, a single stable reference gene is almost non-existent [42, 43]. Previous studies have shown that different genes are persistently expressed in different species under different conditions [44–47]. Therefore, each species requires the most appropriate reference gene for analyzing unique sample types and experimental conditions. To date, there is no report on the most stable reference gene for normalizing gene expression in *Kobresia*. Thus, this study analyzed 12 candidate reference genes for the expression of *K. littledalei* under different conditions. The results revealed different reference genes across different tissues and conditions, consistent with Duan et al. [48] and Wang et al. [18]. For example, *ACTIN* and *GAPDH* were ideal for comparing different treatments in leaves, *ACTIN* and *RPL6* were most suitable for leaves under mannitol stress, *SOD* and *RPL6* were more suitable for root tissues under mannitol treatment, and *EF1-alpha* was suitably used to compare leaves under ABA and GA treatments, respectively.

This study used five analytical methodologies, Delta-Ct, geNorm, NormFinder, BestKeeper, and RefFinder, to estimate the expression stability of internal reference genes in different tissues during drought, salt, ABA, and GA treatments. The purpose was to circumvent the limitations of using a single algorithm analysis. The first four methods were used to evaluate the expression stability of candidate genes, and RefFinder calculated the final ranking. This reference gene ranking varied across the five algorithms for the same set of experimental data. For example, in mannitol-stressed leaves, Delta-Ct, BestKeeper, geNorm, and RefFinder proposed *ACTIN*, but NormFinder suggested *RPL6* as the suitable reference gene. The results are similar to reports in *Hylocereus undatus* [49], *Toona ciliate* [42], *Salsola ferganica* [50], *Prunus persica* [51], *Miscanthus sacchariflorus* [11], *Schima superba* [52], and *Fragaria ananassa* [53]. The differences might be due to the discrepancies in the algorithms.

In brief, the variation measurements were used to determine the stability of gene transcription with the geNorm and NormFinder methods. The pairwise correlation using geNorm is successful for small sample sizes but is biased towards picking genes that are mutually associated with each other. The NormFinder model-based method requires higher sample sizes than geNorm (> 8) and discriminates between within-group and inter-group variances. Therefore, NormFinder is suitable for identifying candidate genes from different sample groups [54]. Additionally, geNorm can determine the optimal number of required reference genes. If the Vn/*n* + 1 is below the threshold (0.15), the advantage of utilizing another (*n* + 1) reference gene becomes restricted. In this study, pairwise variation analysis revealed that the V2/3 value of the two most stable reference genes was < 0.15 across all tissues and conditions, indicating that these reference genes were sufficient for normalizing the gene expression in *K. littledalei*.

Another method, the BestKeeper analysis, uses the correlation between the Cq and an index derived from the geometric mean of the candidate. Thus, a threshold value of SD (1.0) was considered for evaluating the stability of the reference in this method. The reference gene was considered stable if the value was < 1.0. In this study, there was at least one constant candidate gene across all samples and treatments, except the combination of all tissues under NaCl, which had one reference gene (*GAPDH*). In contrast, the combination of all tissues under GA treatment (*GAPDH* and *28 S*) and all samples had *GAPDH* and *ACTIN*, respectively. Nevertheless, the ranking order was still acceptable because the expression stability is a relative concept. This cutoff is a rule of thumb; the threshold is not fixed, symbolizing the stringency for picking the reference gene [14, 55].

In this study, RefFinder was used as the final ranking algorithm. This tool selected *ACTIN* as the most stable reference gene in all treatments and tissues, followed by *RPL6*. *UBQ* was the most unstable reference gene. RefFinder uses the rankings of the candidate genes across these different algorithms to attribute an ordinal “weight” for each candidate gene. The final rankings are then computed as the geometric mean of the weighted rankings [56, 57]. Thus, numerous studies have commonly used this algorithm to validate reference genes [14, 41, 58]. RankAggreg, the other important tool for calculating the final ranking of selected reference genes from other algorithms, uses a cross-entropy Monte Carlo or genetic algorithm to produce aggregated ordered lists based on rankings [42, 59].

Despite their slightly different rankings, all five algorithms determined *ACTIN* as the best reference gene for most tissue samples and treatments. *ACTIN* was among the first reference genes used in gene expression quantification and remains one of the most used internal standards today [60]. The *ACTIN* mRNA encodes a ubiquitous cytoskeleton protein that participates in diverse physiological eukaryotic activities, such as plant and organ development, vesicle and organelle movement, and cell signaling transduction [61]. The second most stable reference gene was *GAPDH*, which encodes the glyceraldehyde-3-phosphate dehydrogenase, an enzyme involved in glycolytic processes. Due to its abundance and extensive expression, *GAPDH* is frequently utilized as an internal control in RT-qPCR [62].

In *Poa pratensis*, *RPL* was the most unstable reference gene in drought-treated leaves [63]. However, *RPL6* had a relatively high ranking in the mannitol and NaCl stresses in this study, consistent with other studies [64, 65]. *RPL6* belongs to the family of RPL that codes for the ribosomal protein large subunit protein, which plays an important role in cellular processes. Meanwhile, the aerenchyma structures of the *K. littledalei* in the alpine swamp meadow have developed to ameliorate the low oxygen stress from soil waterlogging or flooding [66]. This adaptation explains why *EF1-alpha* demonstrated high stability in leaf tissue following the ABA and GA treatments, similar to the results of previous research [67, 68]. The polyribosomal protein coded by the *EF1-alpha* gene participates in ribosomal structure and biogenesis [69]. Saraiva et al. [70] reported that *EF1-alpha* undergoes expression diversification in response to hormone exposure and that *EF1α 3* is predominantly found in aerial tissues.

Furthermore, *UBQ* was the most unstably expressed reference gene in most samples, implying that it is unsuitable for *K. littledalei*, consistent with the results of Zhao et al. [71]. However, *UBQ* was the ideal housekeeping gene for studies of *Oryza sativa* [72], *Miscanthus lutarioriparia* [73], *Magnolia × soulangeana* [74], and *Boehmeria nivea* [75].

We performed a relative gene expression study of *BSK* and *AP2/ERF*, implicated in diversion plant processes, to check the validity of selected reference genes [6, 76]. The expression of *BSK* and *AP2/ERF* was analyzed in various *K. littledalei* tissues (leaf, stem, and root) subjected to abiotic (mannitol and NaCl) stress for various durations (0, 2, 6, 9, 12, 24 h). We normalized the data by comparing the outcomes of the top-two-ranked genes alone and combined and the most unstable reference gene. Further, normalization using the top-two-ranked genes produced a similar expression trend to each individual stably expressed target gene, consistent with the combination of stably expressed reference genes under mannitol and NaCl stress. There were subtle differences in expression levels, indicating that using the top two target genes (in combination) for normalization could further reduce the errors caused by a single reference gene [14, 77]. Similarly, Škiljaica et al. [77] showed that combining the unstable reference gene with one or two top-ranked genes for normalization may substantially mitigate the inaccuracy generated by a poorly chosen reference gene. Meanwhile, the lowest-ranked genes overestimated the relative gene expression of *BSK* and *AP2/ERF*.

Several studies have focused on the stability of candidate reference genes in single plant tissue responses to multiple stresses or treatments [50, 78, 79]. Nonetheless, the findings of the present study highlight that these studies may have overlooked the differences in the stability of the reference genes in different tissues exposed to the same stress or treatment. Under specific conditions (mannitol and NaCl stress), the reference genes selected from each unique tissue showed higher trend and relative expression accuracy of the target genes than reference genes selected from a wider range (e.g., all leaf samples, all stem samples, all root samples, and all samples). This result is consistent with the Yin et al. [13] and Wang et al. [14] findings.

Therefore, before analyzing gene expression under different experimental conditions and plant tissues, it is necessary to evaluate and confirm the reference genes to ensure the reliability of the stable reference genes. The single-use of general reference genes screened from a broader range of target gene expression patterns is less effective than the reference genes screened from specific plant species, living environments, and test tissue. However, it is undeniable that the single use of total general genes can obtain relatively reliable results under some treatments or conditions. The relative expression levels of target genes varied with the difference and number of selected reference genes [13]. Thus, target reference gene use might be a quick, convenient, and time-saving method for analyzing the relative expression of genes using stable reference genes selected from a broader range to minimize the errors caused by the single use of a total general reference gene. Furthermore, the selected reference genes, *ACTIN* and *GAPDH*, showed high stability in cold-stressed *Kobresia* plants, indicating that they could be used as relatively stable reference genes in the other *Kobresia* species (Data not shown).

Most *Kobresia* plants are polyploid, and the basic chromosome number of *Kobresia* species is 16 to 40 [80]. Thus, the stability of *ACTIN* and other stable reference genes in other *Kobresia* species and conditions requires further verification.

The following gene quantitative investigations should include preliminary tests according to species, tissues, and plant environment variations to identify stable reference genes that properly reflect the relative gene expression. Furthermore, future molecular research involving *Kobresia* plants under abiotic stress and hormone treatments will ensure the correctness of the RT-qPCR normalization.

## Conclusion

This study presents the first systematic and exhaustive analysis of potential reference genes in the *Kobresia* plants (*K. littledalei*), validating the accuracy of the selected reference genes. The most stable reference genes were distinct in different tissues and under abiotic/hormone stresses. The combined stability of the two reference genes was better than that of the single ones. Under normal conditions, *ACTIN* was the most stable reference gene in the leaf and stem tissue, and *GAPDH* in the root tissue. Under mannitol stress, *ACTIN* was the most stable reference gene in the leaf tissue and *GAPDH* and *SOD* in stem and root tissues. Further, *RPL6* was the most stable reference gene in the leaf and stem tissues, and *GAPDH* in the root tissue under NaCl stress. Under ABA treatment, *EF1-alpha* was the most stable for leaf tissue, and *SOD* and *ACTIN* in the stem and root tissues. Finally, *EF1-alpha* was the most stable reference gene under GA treatment in the leaf, and *RPL6* and *28 S* in the stem and root tissues, respectively.

### Supplementary Information

**Supplementary Material 1.**

**Supplementary Material 2.**

## Data Availability

All data generated or analysed during this study are included in this published article (and its supplementary information files).
