# Supplementary material for: Identification and validation of stable reference genes for RT-qPCR analyses of Kobresia littledalei seedlings
Source: BMC Plant Biol. 2024 May 11;24:389. doi: 10.1186/s12870-024-04924-w (PMC11088182; doi:10.1186/s12870-024-04924-w)
Supplement: Supplementary file 2 — Supplementary Material 2. [file 12870_2024_4924_MOESM2_ESM.docx]

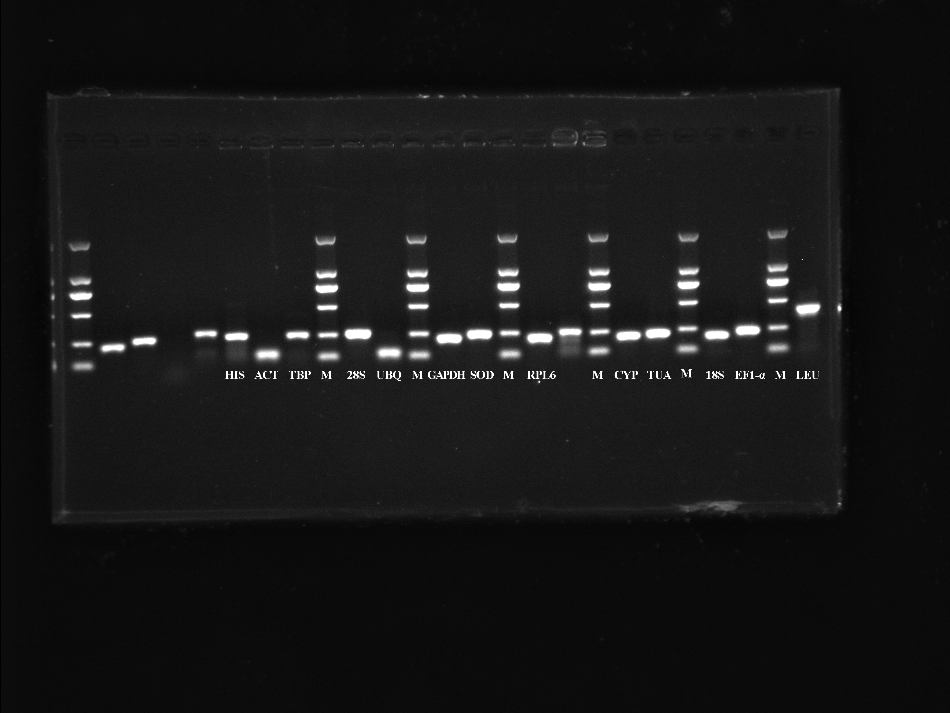


**Supplemental Figure S1.** The full length original and uncropped gel of 13 candidate reference genes.
